# Antimicrobial residue occurrence and its public health risk of beef meat in Debre Tabor and Bahir Dar, Northwest Ethiopia

**DOI:** 10.14202/vetworld.2018.902-908

**Published:** 2018-07-07

**Authors:** Birhan Agmas, Mulugojjam Adugna

**Affiliations:** 1 Department of Veterinary Science, College of Agriculture and Environmental Science, Bahir Dar University, Bahir Dar, Ethiopia; 2 Department of Biology, Faculty of Applied Science, Debre Tabor University, Debre Tabor, Ethiopia

**Keywords:** antimicrobial residue, beef, Ethiopia, with drawl time

## Abstract

**Background and Aim:**

Antimicrobial residues are the parent compounds, their metabolites, and associated impurities of veterinary drugs in any edible portion of an animal product. It can result in severe consequences in human if it is consumed concentration level higher than the standard residue limits. This study aimed to determine the prevalence and its public health risk of antimicrobial residue in fresh beef meat at Bahir Dar and Debre Tabor towns, Northwest Ethiopia.

**Materials and Methods:**

A cross-sectional study was conducted from June to November 2017. The data were collected through interview questionnaire survey and laboratory experiment using Premi^®^(R-Biopharm, Germany) test Kit. Data were entered; analyzed using SPSS version 20.0.

**Results:**

The result of this study showed that almost all beef farms 42 (97.67%) were using tetracycline (oxytetracycline). In addition to β-lactams, (pinstripe) 21 (48.84%), and sulfonamides drugs including sulfadimidine and diminazene aceturate each 4 (9.30%). No one beef farm has respected drug withdrawal period and lack of awareness about antimicrobial side effects in 37 (86.05%) farms. Of 250 beef cattle slaughtered, antimicrobial residue positivity were 191 (76.4%) giving a 95% confidence interval of 71.10-81.70%. Origin of beef farm system was not significantly associated (p>0.05) with antimicrobial residue positivity.

**Conclusion:**

Prevalence of antimicrobial residue in beef meat in Bahir Dar and Debre Tabor towns were high and also the drug residue detected was higher than the standard level. It implies that; it has the public health hazard.

## Introduction

The beef-fattening industry is the fastest means of ameliorating the protein deficiency in Ethiopia. The high turn-over rate, job opportunity for youth, considered as respected gust meat in the country culture and one that solves the malnutrition problem have given more credence to beef fattening among livestock farming. Due to these reasons, the need to meet up the demand for beef meat has necessitated the large-scale production of beef subsequent use of veterinary drugs, especially antimicrobials [1]. These antimicrobials/the parent compounds and/or their metabolites are tending to accumulate in tissues to form residues at different concentrations [2,3].

Antimicrobial residues are unwanted chemicals which have persistence ability through the food web and potential negative impacts on humans [2,3]. Antimicrobial drugs are used widely to improve health, growth promotion, feed efficiency, and to reduce the incidence of diseases. However, they are implemented improperly due to the lack of appropriate knowledge about their dose, withdrawal time and side effects; when used without veterinarian supervision [3,4]. Although antimicrobial benefit most of its uses, the illegal and frequent use of these drugs has led to the accumulation of hazardous antimicrobial residues in edible animal origin foods destined for human consumption then result in the public health hazard.

Human acquires the risk by ingesting antimicrobial residue in meat, milk, eggs those have residue level higher than maximum residue limits (MRLs) and acceptable daily intake [4,5]. The immediate effect of antimicrobial residue is allergenicity and toxicity in human through the food chain [3,4]. The long-term health adverse effects such as increased likelihood include disruption of normal human flora in the intestine (microbiological effects), carcinogenicity, and teratogenicity [3,5]. Other drug residue problems are the development of antibiotic-resistant microbes and drug misuse [3,4,6,7].

The presence of antimicrobials is detected in animal products by screening methods and chromatographic techniques. The screening method is generally performed by microbiological, enzymatic, and immunological methods [8,9]. Premi^®^(R-Biopharm, Germany) test kit is one of a rapid microbiological screening test. It is a commercially available agar diffusion test based on the principle of growth inhibition of microorganisms [10]. The Premi^®^ test is convenient, easy to use, and suitable as an initial screening test for antimicrobial residues. It also has high specificity (95.3%) and very satisfactory sensitivity (72.5%) compared to the gold standard [10,11]. Hence, it is used in remote areas in developing countries [11].

Level of drug residue have the limit, but in developing countries has not yet respect this level in foods of animal origin [12]. In Ethiopia; currently, beef fattening farms use drugs irrationally to fatten. In spite of this frequent and overdose use of drugs, studies about residue burden are limited. Nevertheless, its public health risk and the occurrence of beef meat are unknown adequately. According to the best of our knowledge, very few studies have yet been conducted in Ethiopia to assess the antimicrobial residue in beef meat; none has been conducted in our study areas. Therefore, this study aimed to identify the antimicrobial residues burden and its public health risk of beef meat in Bahir Dar and Debre Tabor towns, Northwest Ethiopia.

## Materials and Methods

### Ethical approval

The study protocol was reviewed and approved by the Institution Review Board of Debre Tabor University. Official permission was also obtained from the respective bodies at Amhara National Regional state livestock resources development promotion Agency (Ref.No 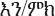/173/21A).

### Informed consent

Verbal informed consent was obtained from each study participant /farm owner/ after being informed about the study for questioner survey.

### Study area

This study was conducted in Debre Tabor and Bahir Dar towns of Northwest region of Ethiopia; from June 2017 to November 2017. Debre Tabor is located at 653 km Northwest of Addis Ababa and a latitude of 11.85000 North; longitude 38.01670 East and an altitude of 2706 meters above sea level. The mean annual rainfall of the town is 866 mm. The mean annual temperature ranges from 15°c to 18.5°c, respectively and a relative humidity of 61.3%. Bahir Dar is located at 556 km Northwest of Addis Ababa at a latitude of 11.60000 North; longitude 37.38330 East and an altitude of 1788 m above sea level. It has a temperature range of 20.2°C-26.9°C [13].

### Source population

The source populations were all beef cattle found in and around Debre Tabor and Bahir Dar towns. The study population includes all beef products those are present in these towns during the study period. The sampling units were those beef cattle’s that included in the study by chance, and the investigator takes the sample on it.

### Sample size and sampling

Forty-three beef farm owners and/attendants were selected randomly in Bahir Dar and Debre Tabor towns for questioner survey. The required sample size for the laboratory was determined by using the following assumptions; the previous study conducted in Debrezite, Ethiopia showed antimicrobial residue occurrence of 82% [14], with 95% confidence level, an error of 5% and 10% add for expected uncertain reaction. Hence, the minimum sample size calculated with single population proportion formula Daniel [15] gives 250 beef cattle. Systematic random sampling technique was carried out to select beef meat in slaughterhouses. The samples were taken in the two study areas proportionally to the number of cattle slaughtered during the study.

### Study design and methodology

The cross-sectional study design was carried out from June 2017 to November 2017. The antimicrobials include in this study were commonly used antibiotics and antiprotozoal drugs in Ethiopia as depicted by Beyene *et al*. [16]. Antibiotics, mainly tetracycline and β-lactam groups; the antiprotozoal drugs; sulfonamide drugs (sulfadimidine and diminazene aceturate) were commonly used in beef farms [16].

### Data collection procedures

The data were collected through interview questionnaire survey and laboratory experiment.

#### Questionnaire survey

A structured questionnaire was developed, and informants (farm owners and attendants) were interviewed. Antimicrobial drugs used as treatment and prophylactic improvement of the health of their animals in farms and the awareness level of the farmers about drug withdrawal period and drug residues were assessed. The questioner also includes information about: the presence of nearby industries, the way of waste disposal, feed and chemical stores and/or other possible conditions that were thought to be the risk of antimicrobial contaminations.

#### Beef meat sampling

Two hundred fifty beef cattle’s were sampled at municipal slaughterhouses. Meat samples of uniform size were collected to avoid the possible error due to size differences. The samples were labeled with an identification number and origin of that beef farm system. The required part and size were collected and stored using 4% formalin, in ice box then sending for inhibition test and analysis. Samples of meat of beef were transported to Amhara regional diagnostic laboratory center then processed.

### Equipment and reagents

The Premi^®^ test is a microbial screening test for the detection of antibiotic residues in food. It is based on the growth inhibition of *Bacillus stearothermophilus*, a thermophilic bacterium that is highly sensitive to many antibiotics and sulfonamide compounds. Assay results are available within 4 h, and the use of spores instead of vegetative cells allows prolonged shelf life of the kit, making it’s commercial distribution feasible [11].

Premi^®^ test kit detects when the presence of drug residue is greater than the drug-specific quantity. For instance in the common drugs used in our study area were oxytetracycline, pinstripe, and sulfonamide drugs detects at 100 µg/kg, 5 µg/kg and 100 µg/kg concentrations in fresh beef meat samples, respectively Table-1 [11].

**Table-1:** Premi^®^ Test detection limits in different animal food products.

Substances	Chicken	Pork	Beef	Eggs	Shrimp
β-lactams					
Amoxicillin	5	5	5	5	15
Ampicillin	5	5	5	5	
Penicillin-G	2.5	2.5	2.5	2.5	5
Cloxacillin		>100		100	
Oxacillin		100			
Dicloxacillin					
Cephalosporins					
Cefquinome	75	100	100		
Ceftiofur	100	200	100	400	
Macrolides					
Tylosin	50	25-50	50	50	
Erythromycin	100	100	100	50	100
Lincomycin	100	100	100		
Tilmicosin	50	50	50		
Spiramycin	1000	1000	1000		
Tetracyclines					
Chlortetracycline	100	100	100	600	1000
Oxytetracycline	100	100	100	400	100
Doxycycline	100	100	100	200	
Tetracycline		50		200	
Demeclocycline		50			
Sulfonamides					
Sulfamethazine	75	50-100	100	25	
Sulfadiazine	75	50-75	75	25	50
Sulfamethizole		50-100			
Sulfguanidine	<200	150	<200		
Sulfadimethoxine		25-50	<100		50
Sulfapyridine	<50	50	<100		
Sulfamethoxypyridine	<100	25			
Sulfisoxazole	<100	25			
Sulfathiazole	<100	25			
Sulfachloropyridazine	<100	25			
Sulfamerazine	<100	25	<100		
Sulfanilamidee	<100	150			
Sulfaquinoxaline	<100	50	<50		
Sulfametiozole	<100		<50		
Sulfamethoxazole				25	
Aminoglycosides					
Gentamicin	100	100	100	100	
Streptomycin	1500	1500	3000	1000	
Neomycin	300	300	300	300	200
Spectinomycin			5000		
Chinolon					
Oxolin acid					>10000
Enrofloxacin	>600	>600	>600		
Flumequine	>100	>100	>100		
Polypeptide					
Virginiamycin	500	500	500		
Bacitracin	500	500	500		
Zn-bacitracin	1250				
Colistin	>1000				
Ionophores					
Salinomycin	1000				
Monensin	1250				
Lasalocid	10000				
Oligosaccharides					
Avilamycin	>5000				
Andere					
Florfenicol	100	100	100		5000
Chloramphenicol	2500	2500	2500	2500	
Trimethoprim	50				
Narasin	1250				
Amprolium	>2000				
Phosphomycine	>1500				
Ronidazole					>5000
Furazolidone	>1500				

All detection limits are given in mg/kg=ppb. Detection limits for other matrices are available on request. Source [11]

### Operational definition

The MRLs; is the maximum amount of the drug residue which is found in food substances that will not cause any health effect or hazard [17]. Acceptable daily intake: Is the amount of a substance that can be ingested daily over a lifetime without appreciable health risk. Antimicrobials are natural products of a microorganism or identical synthetic products or similar semi-synthetic products that inhibit the growth of or destroy microorganisms [18].

### Quality control and quality assurance

A standard operating procedure was strictly followed for sample collection, transport, and storage. Special emphasis was given to coding the sample. All reagents that use for testing were checked for their shelf life and being an appropriate temperature before using them. Test procedures were followed according to the manufacturer’s instruction [11].

### Data management and analysis

Data obtained from the laboratory tests and questionnaires were entered and analyzed using the statistical software SPSS version 20. Descriptive analysis of the collected data was done for most variables in the study using statistical parameters such as percentage and mean. The association was identified by 95% of confidence interval and p-value. The p<0.05 were considered as statistically significant.

## Results

### Antimicrobial drugs used in selected beef farms

A total of 43 beef farms were interviewed and shown that all beef farms use three or more antimicrobial drugs. This research result revealed that almost all beef farms 42 (97.67%) were using tetracycline (oxytetracycline). In addition to β-lactams, (pinstripe) 21 (48.84%) and sulfonamides drugs including sulfadimidine and diminazene aceturate each 4 (9.30%) (Table-2).

**Table-2:** Antimicrobial drugs used in selected beef farms.

Name of chemicals and drugs	Beef farm use of drugs

Antibacterial agents	Frequency (%)
Tetracyclines	
Oxytetracycline	
Yes	42 (97.67)
No	1 (2.33)
β-lactams	
Penstripe	
Yes	21 (48.84)
No	22 (51.16)
Sulfone amides	
Sulfadimidine	
Yes	4 (9.30)
No	39 (90.70)
Diminazene aceturate	
Yes	4 (9.30)
No	39 (90.70)

### Antimicrobial residue risk factors associated with poor practice

In these study areas, 21 (48.84%) of the selected beef farms were using non-professionals to manage drugs for their beef cattle, but the remaining 22 (51.16%) beef farms were using animal health workers to administer drugs. No one beef farm has respected drug withdrawal period due to lack of awareness about antimicrobial side effects 37(86.05%) (Table-3).

**Table-3:** Antimicrobial residue risk factors associated with poor practice.

Variables	Frequency (%)
Professional	
Veterinarian	7 (16.28)
Animal health assistance	15 (34.88)
Non-professional	21 (48.84)
Awareness	
Aware	6 (13.95)
Non aware	37 (86.05)
Respecting withdrawal period	
Yes	0
No	43 (100)
Drug choice	
Cost	3 (6.98)
Availability	39 (90.69)
*Other	1 (2.33)

Other: Indicates user-friendly, antimicrobial effect, cellular activity, margin of safety

### Drug residue positivity in Premi^®^ test kit

**Figure-1:**
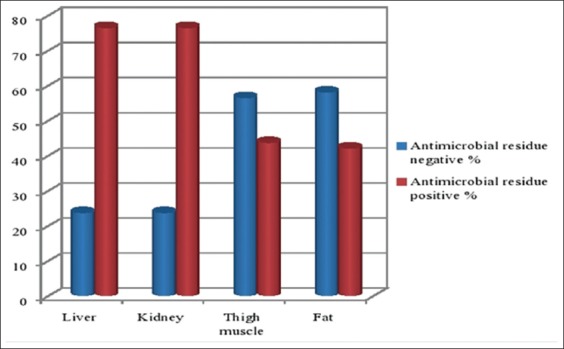
Presence of antimicrobial residues in different tissues of beef meat.

Out of 250 slaughtered cattle; tested for the presence of antimicrobial residue under Premi^®^ test kit; 191 (76.4%) of giving a 95% confidence interval of (71.10-81.70%) showed a positive result. Premi^®^ test kit positivity of antimicrobial drug residues in different organs of beef meat in the study areas was varying. The highest frequencies of these residues were recorded in the liver and kidney 191 (76.4%) in both organs while minimum frequency detected in fat 105 (42%), (Figure-1).

### Origin of beef cattle and residue positivity

The origin of beef cattle system was recorded during sampling; 115 beef cattle originated from smallholder farmers, and 135 beef cattle are from the semi-intensive farm system. The test positivity was 86 (74.78%) in the smallholder and 105 (77.78%) in semi-intensive farms. The farm system was not significantly associated (p>0.05) with antimicrobial residue positivity in beef meat. Antimicrobial residues occurrence and origin of beef farm system p=0.241 when subjected to Chi-square analysis.

## Discussion

Results from this study showed that the highest prevalence 191 (76.4%) of giving a 95% confidence interval of (71.10-81.70%) antimicrobial residue in beef meat detected by Premi^®^ test kit in Bahir Dar and Debre Tabor towns. Of those 250 beef cattle slaughtered and sampled, 191 (76.4%) were from the liver and kidney in both, 109 (43.6%) from thigh muscle, and 105 (42%) from fat. This study finding was much higher than other African countries antimicrobial residue records in beef meat such as Nigeria 54.44%, Kenya 45.6%, Ghana 30.8%, and Sudan 17.33% [19-22]. This study has the similar high result with studies that conducted on antimicrobial residue test on the beef meat samples in central parts of Ethiopia; Addis Ababa, Debre Zeit, and Nazareth slaughterhouses, (93.8%), (37.5%), and (82.1%) were tested positive for oxytetracycline [14].

This high prevalence of antimicrobial residue in these study areas might be due to the irrational utilization of large amount of drugs by farmers. Besides that withdrawal period of the antimicrobial was not respected, use of antimicrobials was not as the prescribed and incorrect route of administration. All of these factors may lead to the contamination of meat by drug residue [3,4]. The questionnaire survey finding of this study also strengths that 43 (100%) of beef cattle farmers were not respected drug withdrawl period, and drugs were given by nonprofessionals 21 (48.84%); due to lack of awareness 37 (86.05%) and might be by negligence. Similar questioner-based study on chemicals and drugs residue in dairy farms in Bishoftu and Modjo, Central Ethiopia showed that 23 (67.6%) have no awareness about drug withdrawal period [16].

In this study origin of beef cattle, the farm system was not significantly associated (p>0.05) with antimicrobial residue positivity. This might be due to in both farm systems were similarly using antimicrobials. That is due to lack of awareness and regulation on drug and chemicals residues, lack of clear regulation on controlling antibiotic contamination of the country and evident lack of with drawl time information about antimicrobial residues in animal-derived foods [23].

The most commonly used antimicrobial agents in the beef farms were oxytetracycline injection, which is used in 97.67% of farms. This high prevalence was in agreement with other studies for instance in Algeria; that has been reported tetracyclines 89.09% the most predominantly prescribed antibiotics [24]. In Ethiopia study conducted in Addis Ababa slaughterhouse shows 93.8% positive for oxytetracycline and another questionaries-based study in dairy farms report’s oxytetracycline injection in 85.7% of farms at Beshoftu central part of the country [14,21]. Our study finding was dissimilarly higher than average African country tetracycline’s use reports those represent 41% prescribed antimicrobial [23]. Tetracycline largely used in our study area may be due to their affordability and accessibility 90.69%, a wide margin of safety and broad-spectrum (Mycoplasma, Gram-positive, and Gram-negative bacteria) and intracellular activity of oxytetracycline [25].

Sulfonamides and β-lactams were used to treat both protozoal and bacterial infections and used in 9.30% and 48.84% of beef farms as an antibacterial and antiprotozoal agent, respectively. This finding is higher than other African country findings; for instance, in Nigeria [19] penicillin use was (14%), and an average African country of 18% was reported [23]. Hence, these drugs in our study area have possible to have residue which might be as depicted above due to, failure to observe withdrawal periods of each drug, extra-label dosages for animals and contamination of animal feed with the excreta of treated animals [3,4,26].

### The public health importance of antimicrobial residues in beef meat

The commonly used antimicrobials in the study area are tetracyclines (oxytetracycline), β-lactams (pinstripe), and sulfonamides (sulfadimidine and diminazene aceturate). Premi^®^ test kit detects the antimicrobial residue concentration level higher than the standard acceptable daily intake. As Codex alimentary international food standards [17] MRLs; up to 200 µg/ kg, 50 µg/kg, and 100 µg/kg and also acceptable daily intake of up to 30 µg/kg, 30 µg/kg, and 50 µg/kg for oxytetracycline, pinstripe and sulfonamide drugs, respectively.

In this study, Premi^®^ test kit detection limit was greater than 100 µg/kg, 5 µg/kg and 100 µg/kg concentrations of oxytetracycline, pinstripe, and sulfonamide drugs in fresh beef meat samples, respectively (Table-1) [11]. These shows that except sulfonamides others were detected lower residue than the MRL, but higher than the acceptable daily intake stated by FAO/WHO standard [17,27]. Even though the test did not exactly quantify the level of residue; it detected higher than acceptable daily intake, and higher prevalence in this study areas shows the probability of public health risk. This high-contamination rate of beef meat in the study areas is likely that consumers experience a high risk of exposure to drug residues [16,23,27,28].

## Conclusion and Recommendations

The presence of antimicrobial residue percentages in beef meat was high. The only antimicrobials used in these areas were oxytetracycline, pinstripe, sulfadimidine, and diminazene aceturate. The highest frequently used drug was oxytetracycline. Indiscriminate and irrational use of antibiotics in beef cattle without following withdrawal period may result in unexpected residues in beef meat and could cause serious health hazards to consumers. All efforts including education of beef farm owners about the proper utilization of antimicrobials, side effect of the irrational use of drugs, observance of the withdrawal period, effective surveillance, monitoring and control on the use of veterinary drugs to prevent drug residues in beef meat were recommended.

## Authors’ Contributions

BA designed the study, collected data, performed the statistical analysis and drafted the manuscript. MA participated in the study design, laboratory processing, and manuscript writing. Both authors contributed to the data analysis, read and approved the final manuscript.
